# Housing instability and violence among women who use drugs in Dar es Salaam, Tanzania

**DOI:** 10.1186/s12954-022-00649-x

**Published:** 2022-06-27

**Authors:** Claire Silberg, Samuel Likindikoki, Jessie Mbwambo, Kristin Mmari, Haneefa T. Saleem

**Affiliations:** 1 Department of Population, Family and Reproductive Health, Bloomberg School of Public Health, Johns Hopkins University, 615 North Wolfe Street, Baltimore, MD 21205 USA; 2 Department of Epidemiology and Biostatistics, School of Public Health and Social Sciences, Muhimbili University of Health and Allied Sciences, P.O. Box 65015, Dar es Salaam, Tanzania; 3 Department of Psychiatry and Mental Health, School of Medicine, Muhimbili University of Health and Allied Sciences, P.O. Box 65001, Dar es Salaam, Tanzania; 4 Department of Population, Family and Reproductive Health, Bloomberg School of Public Health, Johns Hopkins University, 615 North Wolfe Street, Baltimore, MD 21205 USA; 5 Department of International Health, Bloomberg School of Public Health, Johns Hopkins University, 615 North Wolfe Street, Room E5033, Baltimore, MD 21205 USA

**Keywords:** Housing instability, Violence against women, Women who use drugs, Opioids, Tanzania

## Abstract

**Background:**

Women who use heroin and other drugs (WWUD) are a key population with elevated risk of physical and sexual violence perpetrated by intimate partners and non-partners. While housing instability has been shown to be associated with violence in high-income settings, this is an underexplored topic in sub-Saharan Africa. In this research, we aimed to assess the relationship between housing instability and various forms of violence within a sample of WWUD in Dar es Salaam.

**Methods:**

This analysis uses data from a parent study from 2018. A total of 200 WWUD were recruited through respondent-driven sampling methods and administered a survey. Two multivariable logistic regression models were built to assess the relationship between housing instability and physical violence (Model 1) and housing instability and sexual violence (Model 2) while controlling for a number of sociodemographic characteristics.

**Results:**

Approximately 35% of participants were classified as housing unstable. More than half of participants (62%) reported experiencing physical violence in the past 12 months, and more than a third (36%) reported sexual violence in the same time period. Housing instability was found to be independently associated with both physical and sexual violence victimization in the past year when adjusting for covariates (Model 1 adjusted odds ratio [AOR]: 2.40, 95% CI 1.22–4.46; Model 2 AOR: 1.93. 95% CI 1.02–3.67).

**Conclusion:**

To our knowledge, this is the first study to document a significant association between housing instability and violence among WWUD communities in sub-Saharan Africa. This analysis adds to the growing body of literature on the relationship between stable housing and livelihood and health outcomes across differing populations. The cyclical nature of housing instability and violence may be disrupted through housing programming that provides safety, security, and stability for WWUD.

## Background

Violence against women continues to be a barrier to disease prevention and mitigation efforts, particularly in populations who experience the dual burden of violence victimization and high rates of HIV and other bloodborne illnesses [1–4]. Women who use heroin and other drugs (WWUD) in Dar es Salaam, Tanzania, are one such population. Recent figures estimate HIV prevalence among WWUD in Dar es Salaam to be as high as 41% as compared to 6.8% among men who use or inject drugs and 4.7% among the general adult population in Tanzania [5, 6]. Needle-sharing, the practice of ‘flashblood,’ and risky sexual behaviors contribute to abnormally high HIV transmission risk within this community [5, 7–9]. ‘Flashblood’ is a form of heroin administration that was first documented among WWUD in Dar es Salaam in which one injects oneself with the blood from someone who recently used heroin [10]. The growing heroin epidemic in Dar es Salaam has fueled public health attention toward new avenues for HIV transmission and the consequences on HIV reduction efforts regionally [3, 11–13]. The heroin epidemic in Dar es Salaam has developed in parallel with the surge in heroin importation into Dar es Salaam over the past 40 years, and there is no evidence to suggest that heroin trafficking in this region is slowing down [14–16]. It is estimated that there will be 150% more people who use illicit drugs in sub-Saharan Africa by 2050 [17].

Violence perpetrated against WWUD is an underexplored topic in communities of people who use heroin and other drugs globally, particularly in the sub-Saharan African context. Global research suggests that WWUD are at heightened risk of physical and sexual violence perpetrated by both intimate partners and non-intimate partners [2, 13, 18]. The relationship between drug use and violence is purported to be bidirectional in nature. Heroin use increases the risk of intimate partner violence (IPV) and other forms of violence victimization, and experiences of violence lead to increased heroin use [19]. One study found that using drugs with intimate partners can increase the risk of IPV more than threefold [20]. In Dar es Salaam, data on violence perpetrated against WWUD are insufficient. However, one study highlights the implications of violence on access to treatment and care. Balaji et al. found that being in a violent relationship is associated with non-enrollment in opioid use treatment [13]. Approximately 83% of WWUD not in treatment reported recently experiencing sexual violence. This figure is more than nine times higher than the national prevalence of women who have ever experienced sexual violence in Tanzania (9%) [21].

Housing instability, and conversely housing stability, is an important environmental determinant of violence and other adverse health outcomes. As shown in cross-sectional research from high-resource settings, there is a significant association between housing instability and physical and sexual violence among women who have experienced IPV [22–24]. Similarly, this association has been established among female sex workers in India and in communities of people who use or inject heroin in Canada [25, 26]. Further, housing instability is linked to risky heroin administration behaviors and is a risk factor for discrete health outcomes. Housing instability is associated with the practice of ‘flashblood’ in Tanzania [9, 26]. Housing instability has also been found to be associated with poor mental health and an increased likelihood of mortality. A longitudinal cohort study on IPV survivors from the USA found that housing instability was as strong of a predictor of poor health as level of danger in an abusive relationship. In this research, women with housing instability were significantly more likely to have negative psychological outcomes and a reduced quality-of-life as compared to women who were not housing unstable when adjusting for relationship danger [27]. Lastly, the relationship between housing instability and adverse outcomes was explored among a cohort of people who use heroin in Vancouver, Canada. The authors found that, after adjusting for participant HIV status and drug use behaviors, such as frequency of heroin use and high-risk injection behaviors, housing instability was independently associated with all-cause mortality [28].

Housing instability expands on the definition of homelessness and includes a range of housing-related behaviors, such as housing transience. It can also encompass an individual’s perception of housing security based on their ability to maintain their current housing status, vis-à-vis economic security or mental health. Housing instability has been defined as frequent evictions, cohabiting in overcrowded or informally shared spaces, ‘doubling up’ with friends, being unable to pay rent, and sleeping outside for extended periods of time [27, 29–31]. Past research has used a dichotomous measure to capture this experience (‘housed’ vs ‘unhoused,’ ‘unstable’ vs ‘stable’), although more recent research used three separate categorical variables to understand different facets of the housing experience (physical location, socio-contextual experience, and perceived stability) [32]. Despite differences in the conceptualization and measurement of housing instability, central to this experience is an individual’s inability to access, secure, and/or maintain a safe and stable living space.

Existing research on WWUD in Dar es Salaam sheds light on housing experiences which are symptomatic of housing instability. One study found that WWUD ‘double up’ with friends, live in shared spaces, and/or live in housing for shorter durations of time [33]. Given the limited research on this topic, the goal of the present analysis is to explore housing instability and the relationship between housing instability and violence perpetrated by a sexual partner among WWUD in Dar es Salaam. We believe that assessing the relationship between housing instability and violence will provide further clarity on the key environmental determinants which impact high HIV transmission patterns and other poor health outcomes within this population.

## Methods

This analysis uses data from a cross-sectional study on WWUD in Dar es Salaam conducted by Johns Hopkins Bloomberg School of Public Health and Muhimbili University of Health and Allied Sciences between November 2018 and February 2019. The study was conducted among women who were 18 years of age or older, reported heroin use in the past 30 days, and resided in Dar es Salaam. We used non-probabilistic respondent-driven sampling methods to recruit women into the study. Community outreach workers identified recruitment ‘seeds,’ defined as women who use drugs familiar with the local communities, who enrolled the first wave of participants. Thereafter, participants were provided with three coupons to recruit peers who met the eligibility criteria. This process was repeated until the desired sample size of 200 participants was reached.

Two research assistants from Muhimbili University of Health and Allied Sciences administered a structured survey tool in a secure, community-based location in the Kinondoni District of Dar es Salaam. The survey included questions related to sociodemographic characteristics, housing, HIV testing and treatment engagement history, drug and alcohol use, heroin injection behaviors, mental health, stigma, social network, and physical and sexual violence. Participants received compensation of 10,000 Tanzanian shillings ($4.32 USD) and a secondary incentive of up to 4000 Tanzanian shillings ($1.73 USD) for each peer recruited into the study.

### Measures

#### Outcome variables and main predictor

The two outcome variables of interest were physical violence perpetrated by a sexual partner in the past twelve months and forced sex perpetrated by a sexual partner in the past 12 months. Physical violence was measured by asking, ‘Within the last twelve months, have you been hit, slapped, kicked, or otherwise physically hurt by a sexual partner?’. Response categories were binary ‘Yes/No.’ Sexual violence was determined by asking, ‘Within the last twelve months, has a sexual partner forced you to have sex?’ Response categories were binary ‘Yes/No.’ The main predictor variable of housing instability was measured by asking, ‘At any time during the past 6 months, have you *not* had a regular place to stay?’ Response categories were binary ‘Yes/No.’

#### Covariates of interest

Sociodemographic characteristics included participant age, educational attainment, number of children, and current relationship status. Participant age (in years) was measured continuously and recoded into three groups: 19–28, 29–38, and 39 + . The highest level of educational attainment was captured as ‘no education,’ ‘primary education,’ or ‘secondary education or higher.’ Current relationship status included the categories of ‘married or living with a partner,’ ‘single,’ ‘non-cohabiting partner,’ and ‘widowed, divorced, or separated.’ Number of children was dichotomized as ‘no children’ and ‘one or more.’

We identified secondary variables for the logistic regression models based on inferred hypotheses from the literature and risk factors associated with violence and/or housing instability in this population [34, 35]. Secondary variables included HIV status, arrest patterns, engagement in transactional sex work, and heroin use behaviors. Participants were asked if they had ever received an HIV test and their current HIV status (‘HIV-positive,’ ‘HIV-negative,’ and ‘unknown’). Additional covariates included ever been arrested (‘Yes/No’) and an arrest within the past 6 months (‘Yes/No’). Heroin use duration was measured continuously and recategorized as ‘ ≤ 1 year,’ ‘2–9 years,’ and ‘10 + years.’ Frequency of heroin use in the past month was assessed with a five-item scale: ‘did not use in past month,’ ‘used every day in past month,’ ‘used a few times per week,’ ‘used once per week,’ and ‘used less frequently than once per week.’ Due to frequency of heroin use in the sample, this variable was recoded into ‘used every day in past month’ and ‘used less frequently than every day.’ Lifetime engagement in transactional sex work (i.e., having ever had sex with someone in exchange for food, money, or shelter) was measured through a binary ‘Yes/No’ response.

### Data analysis

We performed independent chi-squared tests and *t* tests to test statistically significant differences between independent variables and predictor variables. Statistical significance was defined as an alpha p value of less than or equal to 0.05. We built two logistic regression models to model the relationship between violence and housing instability while adjusting for key sociodemographic information and arrest patterns, transactional sex work, heroin use behaviors, and HIV status. Regression models were built using an iterative analytical process based on the best subsets variable selection method [36]. All relevant variables were included in the models; final variables were selected based on the most parsimonious model as defined by Akaike information criterion (AIC). ‘Educational attainment,’ ‘current relationship status,’ and ‘children’ were removed from models due to non-significance. Furthermore, ‘lifetime history of arrest’ and ‘recent arrest’ were dropped due to collinearity with ‘transactional sex work.’ The final multivariable logistic regression models (Tables 2 and 3) were checked using a Hosmer–Lemeshow goodness-of-fit test. All data were analyzed using Stata v17.0.

## Results

Sample statistics stratified by housing status are presented in Table 1. The mean age of participants was 33.5 years old. The majority of participants reported experiencing physical violence perpetrated by a sexual partner in the past 12 months (62%) and slightly more than a third (36%) reported a forced sexual encounter in the same time period. Approximately 35% of the sample was classified as housing unstable within the past 6 months. In terms of current relationship status, 37% of women reported being married or living with a partner, 26% reported being in a non-cohabiting relationship, 29% of participants reported not being in a relationship, and 15% reported being divorced, widowed, or separated. Approximately 85% of participants reported using heroin every day in the past month. The average duration of heroin use was 6 years. A small proportion of women reported having ever injected heroin (13%). The vast majority of participants had engaged in transactional sex (85%) and had exchanged sex for drugs (83%). Approximately 81% of women reported having ever been arrested and 60% of participants had been arrested within the 6 months prior to being surveyed. 90% of women had ever received an HIV test. In total, 28% of participants reported being HIV-positive, 62% reported being HIV-negative, and 10% reported an unknown status.

**Table 1:** Full sample characteristics and bivariate results

	Total	Housing unstable	Not housing unstable	p value
	*n* = *200*	*n* = *70*	*n* = *130*	
	Mean or %	Mean or %	Mean or %	
*Demographics*				
Age (Mean)	33.5	32.86	33.86	0.41
19–28	32.5%	32.9%	32.3%	0.94
29–38	45.6%	51.4%	42.3%	0.22
39 +	22.0%	15.7%	25.4%	0.12
Educational status				0.88
No education	6.0%	5.7%	6.2%	0.90
Some primary	80.0%	77.1%	81.5%	0.46
Secondary +	14.0%	17.1%	12.3%	0.35
Relationship status				0.21
Married/live with partner	37.0%	34.3%	38.5%	0.57
Divorced, separated, or widowed	14.5%	11.4%	16.2%	0.37
Non-cohabiting partner	25.5%	22.9%	26.9%	0.53
Not in relationship	29.0%	41.4%	22.3%	0.004**
Number of children				0.27
No children	13.5%	17.1%	11.5%	
1 + Children	86.5%	82.9%	88.5%	
Transactional sex (ever)	85.0%	90.0%	82.3%	0.15
Exchanged sex for drugs (ever)	82.5%	90.0%	78.5%	0.04*
Reported HIV serostatus	28.0%	25.7%	29.2%	0.72
Arrested (ever)	81.0%	92.9%	74.6%	0.00*
Arrested (past 6 months)	59.5%	70.0%	53.8%	0.04*
*Drug use*				
Heroin use every day in past 1 month	84.5%	90.0%	81.5%	0.12
Duration of heroin use (mean number of years)	6.0	6.5	5.6	0.05*
Injection drug use (ever)	13.0%	17.1%	10.8%	0.20
*Violence*				
Sexual violence in past 12 months	35.8%	47.1%	29.6%	0.02*
Physical violence in past 12 months	62.0%	74.3%	55.1%	0.01**

^*^Chi-squared test of difference between groups statistically significant at < 0.05 level

^**^Chi-squared test of difference between groups statistically significant at < 0.01 level

Participants with housing instability were significantly more likely to not be in a current relationship as compared to women who were housing stable (41 vs. 22%, *p* = 0.001). A significantly greater proportion of women who were housing unstable had ever exchanged sex for drugs (90 vs. 79%, *p* = 0.04) and had been arrested in the 6 months prior to being surveyed (70 vs. 54%, *p* = 0.04). Approximately 74% of participants classified as housing unstable experienced physical violence in the past 12 months as compared to 55% who did not experience housing instability (*p* = 0.01). Similarly, 47% of participants classified as housing unstable experienced sexual violence in the past 12 months as compared to 30% of women not housing unstable (*p* = 0.02).

The results from the final multivariable logistic regression models are presented in Tables 2 and 3. In the model for physical violence (Table 2), the odds of experiencing physical violence in the past year was 2.40 times higher among women who experienced housing instability in the past 6 months while holding other variables constant (AOR = 2.40; 95% CI 1.22–4.46). Table 3 shows a similar pattern between housing instability and sexual violence. Housing instability was found to be significantly associated with a forced sexual encounter in the past year (AOR = 1.93, 95% CI 1.02–3.67). Additionally, participants who had ever engaged in transactional sex work were 2.41 times more likely to report having experienced physical violence in the past year (AOR = 2.41, 95% CI 1.03–5.05) and almost four times more likely to report sexual violence in the same time period (AOR = 3.47, 95% CI 1.12–9.89). We found no statistically significant association between physical and sexual violence and participant age, HIV serostatus, or duration of heroin use.

**Table 2:** Final multivariable model assessing relationship between housing instability and physical violence (n = 197)

Variables	Unadjusted			Adjusted		
	Odds ratio	95% CI	*P Value*	Odds ratio	95% CI	*P value*
Housing instability	2.35	1.24, 4.46	0.01**	2.40	1.22, 4.46	0.01*
Age (< 28)	2.56	1.33, 4.76	0.00**	0.48	0.22–1.07	0.07
HIV serostatus	0.85	0.56, 1.30	0.452	0.92	0.58, 1.46	0.74
Heroin duration						
< = 1 year	Ref	Ref	Ref	Ref	Ref	Ref
2–10 years	0.74	0.37–1.50	0.41	0.71	0.32, 1.60	0.41
> 10 years	0.80	0.34–1.91	0.66	0.83	0.30, 2.33	0.72
Transactional sex	2.46	1.11, 5.41	0.03*	2.41	1.03, 5.05	0.04*

^*^Statistically significant difference at < 0.05 level

^**^Statistically significant difference at < 0.01 level

**Table 3:** Final multivariable model assessing relationship between housing instability and sexual violence (n = 193)

Variables	Unadjusted			Adjusted		
	Odds ratio	95% CI	*P Value*	Odds ratio	95% CI	*P value*
Housing Instability	2.11	1.15, 3.90	0.02*	1.93	1.02, 3.67	0.04*
Age	1.36	0.72, 2.55	0.34	0.62	0.28, 1.34	0.22
HIV serostatus	0.94	0.60, 1.47	0.79	0.96	0.59, 1.57	0.89
Heroin duration						
< = 1 year	Ref	Ref	Ref	Ref	Ref	Ref
2–10 years	1.35	0.65, 2.78	0.42	1.29	0.57, 2.93	0.54
10 + years	1.85	0.77, 4.43	0.17	1.88	0.65, 5.41	0.24
Transactional sex	4.31	1.44, 12.93	0.00**	3.47	1.12, 9.89	0.03*

^*^Statistically significant difference at < 0.05 level

^**^Statistically significant difference at < 0.01 level

## Discussion

The purpose of this analysis was to examine the association between housing instability and physical and sexual violence within WWUD populations residing in Dar es Salaam. Results from the analysis are the first, to our knowledge, to document a significant association between housing instability and violence in sub-Saharan Africa. We also report rates of both physical and sexual violence experienced in the past year within this population. Approximately 62% of women reported at least one episode of physical violence in the past year and 36% of women reported a forced sexual encounter. The rate of physical violence reported in this sample is almost twice as high as the global lifetime incidence reported in Tanzania [21, 37]. Similarly, the percentage of women who reported sexual violence in the past year is more than four times higher than the 12-month incidence among the general population [21]. The profound disparities in rates of violence between WWUD and the general population shed light on the omnipresent nature of violence and point to an urgent public health and human rights crisis.

The results from multivariable logistic regression models support the hypothesis that housing instability is independently associated with physical and sexual violence in the past year. Research on IPV survivors has shown that the relationship between housing stability and violence perpetrated by an intimate partner is cyclical in nature. There are multiple potential pathways which may explain this relationship. Women who are economically insecure, discriminated against by housing providers, and/or unable to afford housing are more likely to depend on intimate partners for housing security and may sacrifice their personal safety for housing stability secured through partnership. Women are likely to face periods of housing insecurity immediately after leaving abusive partners. Despite the risk of violence, women will often return back to partners to secure stable housing for themselves and their children [22, 38].

In addition to violence perpetrated by an intimate partner or spouse, WWUD are also likely to experience violence perpetrated by other people who use drugs. WWUD in Dar es Salaam spend time in drug camps (also known as *vijwe* or *dago*), which are male-dominated encampments usually located in discrete spaces such as highways and along railroad tracks throughout Dar es Salaam. Research has found that women face high risk of violence in these settings. Theft, attacks, robbery, and sexual assault are commonplace, and women may be more vulnerable to these incidents if they are unstably housed and choose to sleep occasionally or permanently in these spaces [10, 39].

The bivariate results stratified by housing status revealed that a larger proportion of housing unstable women were single as compared to stably housed women. However, we found that relationship status was not predictive of physical or sexual violence in the past year in adjusted or unadjusted models. These preliminary findings suggest that one’s relationship status does not have any bearing on incidents of violence; both single and partnered women experience similar levels of physical and sexual violence perpetrated by sexual partners. These findings are supported by previous research on WWUD that found that women are at risk of violence from multiple perpetrator sources simultaneously including transactional sex clients, intimate partners, and/or spouses [19, 40]. One study on WWUD from California found that having a long-term intimate partner or spouse was not protective against non-partner violence. In this research, women engaged in sex work regardless of their relationship status and were coerced into sex work by drug-using spouses/partners in order to secure money to maintain partner drug supply [40]. In the present analysis, we found that 85% of participants had ever engaged in transactional sex. Similarly, Williams et al. found in 2007 that 85% of WWUD in Tanzania engage in transactional sex to acquire heroin and to earn an income [33]. Results from our second regression model showed that lifetime engagement in transactional sex was the strongest predictor of sexual violence in the past year. Populations who engage in transactional sex work are vulnerable to violence perpetrated by transactional sex clients and are also at increased risk of HIV infection. Future research is needed in this setting to better understand the circumstances in which violence manifests and the main perpetrators of violence.

Stable housing programs address the risk of violence victimization by physically sheltering women against street perpetrators and providing protection for women who are dependent on abusive partners for housing and other resources [41]. The benefits of secure housing extend beyond violence prevention and violence revictimization. Safe housing programs for violence survivors and other housing unstable populations are grounded in the belief that housing is foundational for tackling other health challenges [42, 43]. Research on ‘housing-first’ interventions has found housing stability to be associated with healthcare services utilization, overdose prevention, HIV treatment adherence, and improvements in mental health [26, 43–45]. One randomized control trial found that housing stability significantly improved mental health and led to greater adherence to ART treatment regimens [45]. Similarly, another study on individuals living with HIV who were housing unstable showed that participants who achieved housing stability were significantly more likely to engage in HIV treatment and 22–26% more likely to achieve viral suppression during the second year of the program [46].

There has been a recent call to action from the scientific community advocating for the scale-up of interventions for people who use drugs that fall outside of the traditional package of harm reduction services recommended by the World Health Organization for populations of people who use or inject drugs [17, 44–51]. The results from this analysis corroborate those efforts. While healthcare services designed specifically for people who use drugs in Dar es Salaam have been offered in recent years, these programs are limited to needle exchange programs and medications for opioid use disorder, particularly methadone maintenance therapy. Community-based sober houses do exist in Dar es Salaam, yet there has been no formal evaluation of the ‘readiness’ of these establishments to respond to the various health needs of WWUD. To our knowledge, there has not been a comprehensive scoping review of service availability for WWUD in Dar es Salaam and an analysis of the gendered distribution of access to housing services. Given the gender-specific health, economic, and social challenges that WWUD face, there is an urgent need for integrated short- and long-term gender-responsive structural approaches to tackle these complex public health challenges. Structural interventions that offer comprehensive and holistic services can serve as potential models for care. A housing-first program that includes the components of physical shelter and integrated harm reduction was launched in 2019 in Tshwane, South Africa, and shows promise [52]. Global interventions that include gender-specific components, such as offering childcare and sexual and reproductive health services, and are ‘women-only,’ have been shown to increase women’s adherence to care and access to services [53, 54]. A community-led ‘safe sanctuary’ model that offers harm reduction services and safety from street violence and is managed by community members may be effective if adapted to the Tanzanian context [55]. Lastly, training healthcare providers and community outreach workers who work with WWUD to screen for IPV should be prioritized immediately. This has shown to be a cost-effective method to help identify survivors of violence and link women to housing services or other supportive programming [56], 57. In order to successfully implement a similar intervention in Dar es Salaam, the capacity of healthcare providers and HIV counselors to facilitate universal violence screenings and refer appropriately in a confident, safe, and rights-based manner needs to be considered.

### Limitations

The results presented are from a cross-sectional survey, so further research is needed to tease out the temporality of the associations. All data were self-reported by participants and may be impacted due to recall error and bias. Variables measure differing time frames, which is a limitation to the relevancy of current behaviors, such as engagement in transactional sex work (ever) and injection drug use (ever). Participants in the study were recruited by a peer-led, non-probabilistic sampling method and are likely to share similar sociodemographic characteristics such as socioeconomic status and regionality. Participants are also likely to share a similar social network. Since the goal of the present analysis was to examine associations between key variables, respondent-driven sampling weighting was not applied. The figures presented in this paper may not be generalizable outside of the sample and should be interpreted with caution.

## Conclusion

This study explores the relationship between housing instability and physical and sexual violence experienced in the past year among WWUD in Tanzania, a growing hotspot for the concurring heroin and HIV epidemics in East Africa. The results from this analysis add to the body of literature on the association between housing status and health and livelihood outcomes among geographically disparate and diverse global populations. Housing instability was found to be independently associated with both physical and sexual violence in the past year when adjusting for age, HIV serostatus, heroin use duration, and engagement in transactional sex. Future research is needed to understand the directionality of this association and the relationship between these risk factors and other health outcomes, including HIV transmission. Safe and stable housing is an instrumental component of violence reduction, harm reduction treatment, and adherence to care and should be offered within the constellation of services designed for WWUD in Dar es Salaam.

## Data Availability

The dataset supporting the conclusions shared in this this article is available upon request.

## Abbreviations

IPV: Intimate partner violence
PWUD: People who use or inject drugs
WWUD: Women who use or inject drugs

## References

[1] Mwangi C, Karanja S, Gachohi J, Wanjihia V, Ngang AZ (2019). Depression, injecting drug use, and risky sexual behavior syndemic among women who inject drugs in Kenya: a cross-sectional survey. Harm Reduct J.

[2] El-Bassel N, Strathdee SA (2015). Women who use or inject drugs: AN action agenda for women-specific, multilevel, and combination HIV prevention and research. JAIDS J Acquird Immune Defic Syndr.

[3] Bowring AL, Luhmann N, Pont S, Debaulieu C, Derozier S, Asouab F (2013). An urgent need to scale-up injecting drug harm reduction services in Tanzania: prevalence of blood-borne viruses among drug users in Temeke District, Dar-es-Salaam, 2011. Int J Drug Policy.

[4] Biomndo BC, Bergmann A, Lahmann N, Atwoli L (2021). Intimate partner violence is a barrier to antiretroviral therapy adherence among HIV-positive women: evidence from government facilities in Kenya. PLOS ONE.

[5] Likindikoki SL, Mmbaga EJ, Leyna GH, Moen K, Makyao N, Mizinduko M (2020). Prevalence and risk factors associated with HIV-1 infection among people who inject drugs in Dar es Salaam, Tanzania: a sign of successful intervention?. Harm Reduct J.

[6] United Republic of Tanzania | UNAIDS [Internet]. 2020 [cited 2021 Oct 4]. Available from: https://www.unaids.org/en/regionscountries/countries/unitedrepublicoftanzania

[7] Khatib A, Matiko E, Khalid F, Welty S, Ali A, Othman A (2017). HIV and hepatitis B and C co-infection among people who inject drugs in Zanzibar. BMC Public Health.

[8] Lambdin BH, Douglas Bruce R, Chang O, Nyandindi C, Sabuni N, Zamudio-Haas S (2013). Identifying programmatic gaps: inequities in harm reduction service utilization among male and female drug users in Dar es Salaam, Tanzania. PLoS ONE.

[9] McCurdy SA, Ross MW, Williams ML, Kilonzo GP, Leshabari MT (2010). Flashblood: blood sharing among female injecting drug users in Tanzania: Flashblood: blood sharing among IDUs in Tanzania. Addiction.

[10] McCurdy SA, Williams ML, Kilonzo GP, Ross MW, Leshabari MT (2006). Heroin and HIV risk in Dar es Salaam, Tanzania: Youth hangouts, mageto and injecting practices. AIDS Care.

[11] Ratliff EA, McCurdy SA, Mbwambo JKK, Lambdin BH, Voets A, Pont S, Maruyama H, Kilonzo GP (2013). An Overview of HIV Prevention interventions for people who inject drugs in Tanzania. Adv Prevent Med.

[12] Williams ML, McCurdy SA, Bowen AM, Kilonzo GP, Atkinson JS, Ross MW (2009). HIV seroprevalence in a sample of Tanzanian intravenous drug users. AIDS Educ Prev.

[13] Balaji D, Mlunde LB, Tran OC, Lambdin B, Mbwambo J, Nyandindi C, Matiko E, Michael Copenhaver R, Bruce D (2016). First report of gender based violence as a deterrent to methadone access among females who use heroin in Dar es Salaam, Tanzania. AIDS Behav.

[14] The Smack Track | The Economist [Internet]. [cited 2021 Oct 4]. Available from: https://www.economist.com/middle-east-and-africa/2015/01/15/the-smack-track

[15] Hurst T (2019). World drug report. Encycl Women Crime.

[16] Degenhardt L, Peacock A, Colledge S, Leung J, Grebely J, Vickerman P (2017). Global prevalence of injecting drug use and sociodemographic characteristics and prevalence of HIV, HBV, and HCV in people who inject drugs: a multistage systematic review. Lancet Global Health.

[17] Donnenfeld Z, Welborn L, Bello-Schünemann J. Drug demand and use in Africa - ENACT Africa [Internet]. 2019 [cited 2021 Oct 4]. Available from: https://enactafrica.org/research/research-papers/drug-demand-and-use-in-africa

[18] Marshall BDL, Fairbairn N, Li K, Wood E, Kerr T (2008). Physical violence among a prospective cohort of injection drug users: a gender-focused approach. Drug Alcohol Depend.

[19] El-Bassel N, Gilbert L, Elwin W, Go H, Hill J (2005). Relationship between drug abuse and intimate partner violence: a longitudinal study among women receiving methadone. Am J Public Health.

[20] El-Bassel N, Wechsberg WM, Shaw SA (2012). Dual HIV risk and vulnerabilities among women who use or inject drugs: no single prevention strategy is the answer. Curr Opin HIV AIDS.

[21] The DHS Program - Tanzania: DHS, 2015–16 - Final Report (English) [Internet]. [cited 2020 Mar 29]. Available from: https://dhsprogram.com/publications/publication-FR321-DHS-Final-Reports.cfm

[22] Baker CK, Cook SL, Norris FH (2003). Domestic violence and housing problems. Violence Against Women.

[23] Pavao J, Alvarez J, Baumrind N, Induni M, Kimerling R (2007). Intimate partner violence and housing instability. Am J Prev Med.

[24] Montgomery AE, Sorrentino AE, Cusack MC, Bellamy SL, Medvedeva E, Roberts CB (2018). Recent intimate partner violence and housing instability among women veterans. Am J Prev Med.

[25] Kennedy MC, McNeil R, Milloy MJ, Dong H, Kerr T, Hayashi K. Residential eviction and exposure to violence among people who inject drugs in Vancouver, Canada. Int J Drug Policy. 2017 10.1016/j.drugpo.2016.12.017 PMC5337171 28104547

[26] Reed E, Gupta J, Biradavolu M, Devireddy V, Blankenship KM (2011). The role of housing in determining HIV risk among female sex workers in Andhra Pradesh.

[27] Rollins C, Glass NE, Perrin NA, Billhardt KA, Clough A, Barnes J (2012). Housing instability is as strong a predictor of poor health outcomes as level of danger in an abusive relationship: findings from the SHARE study. J Interpers Violence.

[28] Zivanovic R, Milloy M, Hayashi K, Dong H, Sutherland C, Kerr T (2015). Impact of unstable housing on all-cause mortality among persons who inject drugs. BMC Public Health.

[29] Frederick TJ, Chwalek M, Hughes J, Karabanow J, Kidd S. How stable is stable? Defining and measuring housing stability. J Community Psychol. 2014

[30] Dickson-Gomez J, McAuliffe T, Convey M, Weeks M, Owczarzak J (2011). Access to housing subsidies, housing status, drug use and HIV risk among low-income U.S. urban residents. Subst Abuse Treat Prev Policy.

[31] Kushel MB, Evans JL, Perry S, Robertson MJ, Moss AR (2003). no door to lock: victimization among homeless and marginally housed persons. Archiv Intern Med.

[32] Dickson-Gomez J, McAuliffe T, Quinn K (2017). The effects of housing status, stability and the social contexts of housing on drug and sexual risk behaviors. AIDS Behav.

[33] Williams ML, McCurdy SA, Atkinson JS, Kilonzo GP, Leshabari MT, Ross MW (2006). Differences in hiv risk behaviors by gender in a sample of Tanzanian injection drug users. AIDS Behav.

[34] Hayashi HD, Patterson TL, Semple SJ, Fujimoto K, Stockman JK (2016). Risk factors for recent intimate partner violence among methamphetamine-using men and women. J Psychoact Drugs.

[35] Rowlands EC, Snyder LM, Boucher AM, Bayoumi M, Marshall Z, Boyd R, LeBlanc S, Tyndall M, Kendall CE (2021). A cross-sectional study of factors associated with unstable housing among marginalized people who use drugs in Ottawa, Canada. PLOS ONE.

[36] Lindsey C, Sheather S. GVSELECT: Stata module to perform best subsets variable selection. Stat Softw Components [Internet]. 2014 Apr 13 [cited 2021 Jun 3]; Available from: https://ideas.repec.org/c/boc/bocode/s457816.html

[37] Clavagnier I. Violence against women. Aide Soignante [Internet]. 2018 [cited 2020 Mar 10];32(200):7. Available from: https://www.who.int/news-room/fact-sheets/detail/violence-against-women

[38] Anderson DK, Saunders DG. Leaving An Abusive Partner: An Empirical Review of Predictors, the Process of Leaving, and Psychological Well-Being. Trauma, Violence, Abus. 2003 10.1177/1524838002250769 14697121

[39] Zamudio-Haas S, Mahenge B, Saleem H, Mbwambo J, Lambdin BH (2016). Generating trust: programmatic strategies to reach women who inject drugs with harm reduction services in Dar es Salaam, Tanzania. Int J Drug Policy.

[40] Lorvick J, Lutnick A, Wenger LD, Bourgois P, Cheng H, Kral AH (2014). Non-Partner violence against women who use drugs in San Francisco. Violence Against Women.

[41] Daoud N, Matheson FI, Pedersen C, Hamilton Wright S, Minh A, Zhang J (2016). Pathways and trajectories linking housing instability and poor health among low-income women experiencing intimate partner violence (IPV): toward a conceptual framework. Women Health.

[42] Tsemberis S, Gulcur L, Nakae M (2011). Housing first consumer choice, and harm reduction for homeless individuals with a dual diagnosis. Am J Public Health.

[43] Clough A, Draughon JE, Njie-Carr V, Rollins C, Glass N (2014). ‘Having housing made everything else possible’: affordable, safe and stable housing for women survivors of violence. Qual Soc Work.

[44] Briggs D, Rhodes T, Marks D, Kimber J, Holloway G, Jones S (2009). Injecting drug use and unstable housing: Scope for structural interventions in harm reduction. Drugs Edu Prev Policy.

[45] Wolitski RJ, Kidder DP, Pals SL, Royal S, Aidala A, Stall R (2010). Randomized trial of the effects of housing assistance on the health and risk behaviors of homeless and unstably housed people living with HIV. AIDS Behav.

[46] Wiewel EW, Singh TP, Zhong Y, Beattie CM, Lim S, Walters S (2020). Housing subsidies and housing stability are associated with better HIV medical outcomes among persons who experienced homelessness and live with HIV and mental illness or substance use disorder. AIDS Behav.

[47] Prins M, Bruneau J (2017). Estimates are not enough: scaling-up interventions to improve the health of people who inject drugs. Lancet Glob Health.

[48] Eric A Ratliff, Sheryl A McCurdy, Jessie KKM, Barrot H. Lambdin, Ancella V, Sandrine P, Haruka M, GPK. An Overview of HIV Prevention Interventions for People Who Inject Drugs in Tanzania. Adv Prev Med. 2013. 10.1155/2013/183187 PMC3549374 23346410

[49] Pinkham S, Stoicescu C, Myers B (2012). Developing effective health interventions for women who inject drugs: key areas and recommendations for program development and policy. Adv Prev Med.

[50] Zamudio-Haas S, Mahenge B, Saleem H, Mbwambo J, Lambdin BH. Generating trust: Programmatic strategies to reach women who inject drugs with harm reduction services in Dar es Salaam, Tanzania. Int J Drug Policy. 2016 10.1016/j.drugpo.2016.01.012 PMC4829444 26880500

[51] Global HIV, Hepatitis and STIs Programmes [Internet]. [cited 2021 Oct 9]. Available from: https://www.who.int/teams/global-hiv-hepatitis-and-stis-programmes/populations/people-who-inject-drugs

[52] Scheibe A, Gloeck N, Madela-Mntla E, Renkin W, Shelly S, Lalla S, et al. 2021 Towards Housing First and Harm Reduction: lessons learnt from addressing opioid dependence and homelessness in Tshwane during the COVID-19 pandemic. South African Heal Rev. 17–28.

[53] Värmå Falk M, Strömdahl S, Ekström AM, Kåberg M, Karlsson N, Dahlborn H (2020). A qualitative study of facilitators and barriers to participate in a needle exchange program for women who inject drugs. Harm Reduct J.

[54] Ayon S, Ndimbii J, Jeneby F, Abdulrahman T, Mlewa O. Barriers and facilitators of access to HIV, harm reduction and sexual and reproductive health services by women who inject drugs: role of community-based outreach and drop-in center 10.1080/09540121.2017.1394965 29067855

[55] Foreman-Mackey A, Bayoumi AM, Miskovic M, Kolla G, Strike C (2019). ‘It’s our safe sanctuary’: experiences of using an unsanctioned overdose prevention site in Toronto. Ontario Int J Drug Policy.

[56] Gilbert L, Jiwatram-Negron T, Nikitin D, Rychkova O, McCrimmon T, Ermolaeva I (2017). Feasibility and preliminary effects of a screening, brief intervention and referral to treatment model to address gender-based violence among women who use drugs in Kyrgyzstan: project WINGS (women initiating new goals of safety): WINGS: an IPV SBIRT model. Drug Alcohol Rev.

[57] Christofides N, Jewkes R (2010). Acceptability of universal screening for intimate partner violence in voluntary HIV testing and counseling services in South Africa and service implications. AIDS Care Psychol Socio Med Asp AIDS/HIV.

